# Reactivation of a Vaccine Escape Hepatitis B Virus Mutant in a Cambodian Patient During Anti-Hepatitis C Virus Therapy

**DOI:** 10.3389/fmed.2018.00097

**Published:** 2018-04-30

**Authors:** Dahlene N. Fusco, Lilia Ganova-Raeva, Yury Khudyakov, Lili Punkova, Aisha Mohamed, Scarlett Se Yun Cheon, Prapti Koirala, Karin L. Andersson, Gonzague Jourdain, Camille Sureau, Raymond T. Chung, Georg Lauer

**Affiliations:** ^1^Medicine/General Internal Medicine and Infectious Diseases, Massachusetts General Hospital, Harvard Medical School, Boston, MA, United States; ^2^Laboratory of Systems Pharmacology, Harvard Medical School, Boston, MA, United States; ^3^Molecular Epidemiology and Bioinformatics Team, DVH/NCHHSTP/CDC, Centers for Disease Control and Prevention (CDC), Atlanta, GA, United States; ^4^Cooper Medical School of Rowan University, Camden, NJ, United States; ^5^Wellesley College, Wellesley, MA, United States; ^6^Gastrointestinal Division, Massachusetts General Hospital, Harvard Medical School, Boston, MA, United States; ^7^Institut de recherche pour le développement(IRD), Marseille, France; ^8^Chiang Mai University, Chiang Mai, Thailand; ^9^Harvard School of Public Health, Boston, MA, United States; ^10^Institut National de la Tranfusion Sanguine INSER U1134, Paris, France

**Keywords:** hepatitis B virus, preS2 deletion, surface antibody escape mutant, HBV vaccine escape mutant, HCV

## Abstract

A 76-year-old Cambodian man co-infected with hepatitis B virus (HBV) and hepatitis C virus (HCV) 6c-1 presented for care. HBV DNA was intermittently detectable despite anti-HBs levels being above the protective threshold. During treatment for HCV, HBV DNA levels increased. Sequencing revealed multiple mutations including vaccine escape mutation and mutations predicted to enhance fitness. This case represents exacerbation of an HBV vaccine escape mutant during a direct-acting antiviral therapy.

## Background

Hepatitis B virus (HBV) reactivation has been reported during hepatitis C virus (HCV) treatment, leading to a need for close surveillance of HBV-infected patients undergoing HCV therapy. We here report a case of HBV reactivation during HCV treatment, with the finding that the reactivated HBV was a vaccine escape mutant.

## Case Report

A 76-year-old man from Cambodia presented for viral hepatitis care. Institutional Review Board approval was waived for this case report. The patient had a medical history of benign prostatic hyperplasia, supraventricular tachycardia, adenomatous polyp of the colon, spinal stenosis, essential thrombocythemia, and hemoglobin E trait. The patient was co-infected with HBV and HCV and had never received treatment for either virus. The patient did not recall any past HBV vaccination. Risk factors for HBV and HCV exposure were unknown. The patient emigrated from Cambodia, where he served in the military. He had no history of blood transfusion, or surgery in Cambodia. He had no tattoos and no history of unsafe injection practices. The patient’s family had no history of liver cancer, liver disease, or cirrhosis. The patient had no history of jaundice or hospitalization for liver disease. On presentation, he had a complaint only of constant generalized weakness. He did not have nausea, vomiting, diarrhea, anorexia, change in bowel pattern, hematochezia, hematemesis, or blood when brushing teeth, rash, arthralgias, unexpected change in weight, abdominal pain, or swelling. Physical examination revealed normal vital signs and no evidence of end-stage liver disease, including no ascites or edema, no appreciable hepatosplenomegaly, no asterixis, no spider angiomata, no jaundice, and clear mentation. Laboratory values revealed creatinine of 1.01 mg/dl, alkaline phosphatase of 43I U/l, ALT of 22 U/l, and AST of 29 U/l. AFP was 3.2 ng/ml. Complete blood count revealed white blood cell count of 5, hemoglobin of 13, and platelet count of 436. HBV DNA 1 month prior to presentation had been 120 IU/ml. The patient had detectable total HBV core antibody (anti-HBc). HBV e antibody and e antigen were not detected. The HBV surface antigen antibody (anti-HBs) titer was at 106 mIU/ml and surface antigen (HBsAg) was undetectable. HBV DNA testing was repeated upon presentation and was reported as <20 IU/ml (HBV DNA not quantifiable). HIV 1,2 Ag/Ab test was negative. HCV genotype was found to be 6c-1, and HCV viral load was 14,500,000 IU/ml. HDV RNA was undetectable. Abdominal ultrasound revealed no suspicious liver lesions and cholelithiasis without cholecystitis. A biopsy was not performed but FibroSure^®^ (LabCorp) blood test indicated F3 fibrosis with bridging. Initial counseling related to HCV and HBV was performed. HCV genotype and HBV viral load were identified for all specimens collected during HCV treatment.

One month later, the patient was seen in follow-up. HCV genotype had been identified as HCV 6c-1.

**Figure 1:**
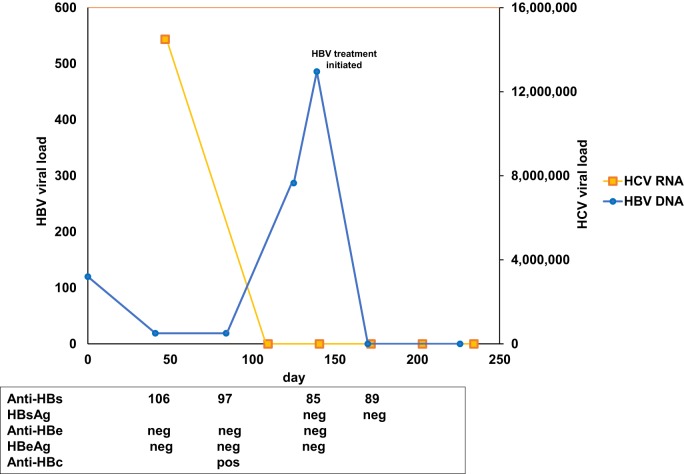
Chronological results for hepatitis B virus (HBV) and hepatitis C virus (HCV) studies. *Y*-axis indicates HBV (left) and HCV (right) viral load over time (*X*-axis). HCV RNA is indicated in yellow, HBV DNA in blue. HBV surface antibody (Anti-HBs), surface antigen (HBsAg), E antibody (anti-HBe), E antigen (HBeAg), and core antibody (anti-HBc) results are presented corresponding to the day of collection.

Based on AASLD guidelines, accessed on September 13, 2016, both sofosbuvir (400 mg)/velpatasvir (100 mg) and ledipasvir (90 mg)/sofosbuvir (400 mg) are acceptable treatments for HCV genotype 6, although the evidence rating was slightly stronger for sofosbuvir (400 mg)/velpatasvir (100 mg). A prior authorization was submitted for sofosbuvir (400 mg)/velpatasvir (100 mg), but rejected by the patient’s insurance with a recommendation to substitute sofosbuvir (400 mg)/velpatasvir (100 mg) with ledipasvir (90 mg)/sofosbuvir (400 mg) 1 pill once daily for 12 weeks and was approved 3 weeks later. Prior to treatment initiation, HBV viral load was found to be <20 IU/ml (Figure 1). One week following treatment initiation, the patient was seen in follow-up. He was tolerating ledipasvir (90 mg)/sofosbuvir (400 mg) well, with improvement of his generalized weakness, and complained only of mild dry mouth. HCV viral load was 19 IU/ml. One week later, the patient was called back to clinic for HBV counseling, as a new black box warning had been added to the ledipasvir (90 mg)/sofosbuvir (400 mg) package insert recommending close HBV monitoring of patients on direct-acting antiviral therapy (DAA). HBV viral load was checked and found to be 287 IU/ml (up from <20 IU/ml 1.5 and 3 months earlier) (Figure 1). HCV viral load was <15 IU/ml. A plan was made to monitor the patient every 2 weeks for clinical symptoms and virologic evidence of worsening HBV, with agreement to present to a clinic earlier if any concerning symptoms developed in the interim (e.g., nausea, vomiting, jaundice, anorexia, or extreme fatigue).

Two weeks later, the patient returned for follow-up and was found to have undetectable HCV RNA and HBV viral load of 486 IU/ml (up from 287 IU/ml) (Figure 1). Due to concern for potential fulminant HBV reactivation while on DAA therapy (1) (Table S1 in Supplementary Material), the patient was initiated on tenofovir therapy 300 mg once daily, with a plan for an indefinite course due to advanced fibrosis. The patient was seen 2 weeks following tenofovir initiation, was doing well without complaints, and was found to have undetectable HBV DNA as well as undetectable HCV RNA. The patient completed a 12-week course of HCV treatment without further event and maintained undetectable HBV DNA and HCV RNA through 12 weeks following the end of HCV treatment at the time of this writing.

**Figure 2:**
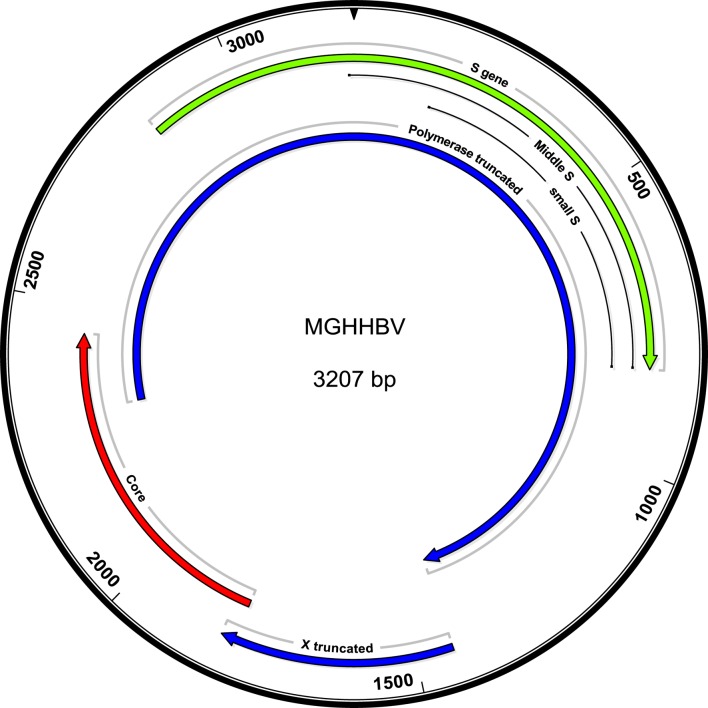
Schematic of the mutant genome. The in-frame deletion is not affecting the ORFS, but shortens pre-S2 and Pol by the corresponding two amino acids. A total of 1,380 truncates the polymerase to 82 amino acids.

Because the patient had exhibited detectable HBV DNA in the presence of HBV surface antibody at the level above the protective threshold, there was concern for the presence of an HBV vaccine escape mutant. Serum from day 139, with HBV DNA 486 IU/ml, was sent for sequencing analysis to the Center for Disease Control, Division of Viral Hepatitis Laboratory. Complete sequence results are presented (Figure S1 in Supplementary Material). The HBV genotype was identified to be C1 (98% identical to published isolate SEA-01, GenBank KM999990). The serological subtype was identified to be adrq+. Eleven mutations were identified (Table 1), including (1) S gene/polymerase: a 6-nucleotide (nt) deletion at nt 45–50 resulting in the removal of amino acids LY from pre-S2 and PI from polymerase, (2) S gene: G145A, a known immune escape S Ag mutation, (3–5) T118M, P120T, P142T: S Ag mutations affecting the a-determinant, (6) polymerase: V173A, (7) nt 1380: frameshift mutation in 50% of reads, (8) X gene: frameshift mutation at 1,581 in 52% of reads, (9) premature stop codon at 1,619, truncating X from 154 to 71 aa, (10) core: basal core promoter mutation A1762T, and (11) core: basal core promoter mutation G1764A. A summary of the mutations and prior literature describing these mutations (Table 1; Table S2 in Supplementary Material) and a schematic of the mutations (Figure 2) are presented.

**Table 1:** Mutations detected in hepatitis B virus (HBV) sequence.

Mutation	Potential effect	Literature
**S gene**		

1. Nucleotide (nt) 45–50 deletion	LY deletion from mid-S2	Sa-Nguanmoo (2) (PMID: 20572086); Gerken (1) (PMID: 1668335)
2. G145A	Vaccine escape	Zanetti (3) (PMID: 2460710), Tong (4) (PMID: 27084035, Sticchi (5) (PMID: 23296324), Rodríguez Lay (6) (PMID: 25978398), Salpini (7) (PMID: 26419862), Aghakhani (8) (PMID: 26384943), Huy (9) (PMID: 16372296), Wang (10) (PMID: 25903946), Amini-Bavil-Olysee S (11) (PMID: 19889778), Avellón (12) (PMID: 16299725), Weber (13) (PMID: 15653412), Lada (14) (PMID: 16501106), Sa-Nguanmoo (2) (PMID: 20572086)
3. T118M	S Ag-mutant affecting a-determinant	Lada (14) (PMID: 16501106)
4. P120T	S Ag-mutant affecting a-determinant	Ye Q (15) (describe P120Q + D144A as vaccine escape mutant) (PMID: 25692622); Lada (14) (describe P120L, P120S) (PMID: 16501106)
5. P142T	S Ag-mutant affecting a-determinant	

**Polymerase**		

1. nt 45–50 deletion	PI deletion from polymerase spacer domain	
6. V173A	Previously reported to restore replicative fitness to LAM mutants (though no LAM mutants detected; no substitution observed at YMDD catalytic domain of Pol)	Ishigami (16) (PMID: 26420956)
7. Frameshift mutation at nt 1,380 (50% of reads)	Truncates polymerase to 82AA, removing 54% of RNase H activity domain, likely rendering the domain nonfunctional	Supports likelihood that mutation + wild type coexist as quasi species, with WT reverse transcripting this mutation. *note: cannot rule out a shift introduced by PCR*

**X gene**		

7. Frameshift at nt 1,380	Causes incorrect start of X gene	
8. Frameshift at nt 1,581 (52% reads)	This frameshift restores the correct frame of X	Bock CT (PMID: 19321929)
9. Premature stop at nt 1,619	Truncates X protein to 71AA instead of complete 154AA	Not previously reported

**Basal core promoter**		

10. BCP mutation A1762T (94% reads)	Decreased HBe Ag expression, Increased HBV replication capacity	Locarnini (17) (PMID: 12616447)
11. BCP mutation G1764A (91% reads)	Decreased HBe Ag expression, Increased HBV replication capacity	Locarnini (17) (PMID: 12616447)
Precore mutation sites T1858, G1896, and G1899 are wild type. Hung (18) (PMID: 22061616).		

## Consent

Written informed consent was obtained from the participant for the publication of this case report.

## Serologic Assays

Hepatitis B virus e Ag/Ab testing was performed using VITROS Immunodiagnostic Products for anti-HBe, HBeAg (http://www.mayomedicallaboratories.com/test-catalog/Performance/8311). For the anti-HBs test, the ARCHITECT AUSAB assay (Abbott Diagnostics) was used. ARCHITECT AUSAB is a chemiluminescent microparticle immunoassay (CMIA) for the quantitative determination of anti-HBs in human adult and pediatric serum and plasma (dipotassium EDTA, lithium heparin, and sodium heparin) and neonatal serum. For the HBsAg test, the ARCHITECT HBsAg qualitative assay (Abbott diagnostics) was used. The ARCHITECT HBsAg qualitative assay is a CMIA for the qualitative detection of HBsAg in human adult and pediatric serum and plasma and neonate serum. For the HBV core Ab test, the ARCHITECT Core assay (Abbott diagnostics) was used. The ARCHITECT Core assay is a CMIA for the qualitative detection of IgG and IgM antibodies to hepatitis B core antigen (anti-HBc) in human adult and pediatric serum and plasma (dipotassium EDTA, lithium heparin, and sodium heparin) and neonatal serum.

## HBV DNA Quantification

Hepatitis B virus DNA quantitative assay was performed using Roche Diagnostics (Cobas AmpliPrep/Cobas TaqMan v2.0).

## HBV DNA Sequencing

Hepatitis B virus DNA was amplified using modified primers (19) that produce a linearized product of the complete genome, followed by random enzymatic shearing, barcoding, and size selection to generate a shotgun next-generation sequencing (NGS) library for the Illumina platform. NGS was done using Illumina v2 150 cycle kit. Reads (150 nt) were assembled by CLC Genomics Workbench (v 10.03, Qiagen, Aarhus) into a complete HBV genome of 3,207 nt at an average coverage of 12,414.78x.

## Discussion

This case represents reactivation of an HBV immune escape mutant containing potential fitness-enhancing mutations during HCV clearance in a non-vaccinated, non-transplant patient. This case is unique for several reasons. First, anti-HBs was detectable simultaneously with HBV DNA, while the patient was negative for HBsAg, HBeAg, and anti-HBe, which is consistent with anti-HBs escape and occult infection. Second, the HBV DNA levels, though detectable within the 3 months prior to treatment, increased from <20 to 400 IU/ml during HCV 6c-1 clearance with DAA, consistent with HBV reactivation due to HCV clearance, as has been reported by others during both DAA and IFN—mediated clearance of HCV (Table S1 in Supplementary Material). Finally, although HBV immune escape mutants have been reported to be of lower or equal fitness compared to wild type (20, 21), the current sequence revealed concurrent immune escape and fitness-enhancing mutations as listed in Table 1.

There are limitations of this case report. The consensus sequence extracted from the NGS data may not correspond to any actual intra-host HBV variant existing in the patient. As for instance, one mutation, or deletion in preS2, may not coexist on the same HBV DNA molecule in the patient with, for example, a frameshift in Pol or X. Deletions 1,380 and 1,581 are found in 50 and 52% of the reads. However, the remaining reads exhibit a wild-type sequence. These data do not allow establishing the *cis*-coexistence of the described mutations. The distance between mutations prevents their identification in one fragment. For the frameshift mutation at nt 1,380 detected in 50% of reads, it is not possible to guarantee that this was not due to PCR error. However, PCR errors of this sort are 100 times less frequent than substitution errors (which happen with about 1.2 × E^4 frequency) and 10 times less frequent than recombination errors. They also tend to occur in homopolymer or short repeat regions and the location nt 1,375 does not contain either.

It is important to note that the sequence described here includes both immune escape and two fitness-enhancing mutations (Table 1). HBV immune escape mutants have been reported to be of lower, equal, and higher infectivity compared to wild type (4, 20–22). While these mutations are frequently described among intra-host HBV variants (23–25), and we have no direct evidence that immune escape and fitness-enhancement mutations occur on the same DNA molecule, their coexistence is nonetheless concerning, given the potential public health consequences of vaccine escape mutants (21). While mutant forms of HBV in cases of occult HBV may theoretically escape detection and could present a risk to blood safety, current methods for blood screening include HBsAg, anti-HBc, and HBV DNA. Although negative for HBsAg, the case described here was positive for anti-HBc and intermittently positive for HBV DNA; therefore, screening would have detected this case through the inclusion of anti-HBc. The greater safety concern for cases such as the one here described relates to the risk of transmission to persons who have been vaccinated (21) and would not be aware of risk related to exposure in the absence of full sequencing. It is not clear how common occult HBV (HBsAg negative/DNA positive) and anti-HBs escape mutations (anti-HBs positive/DNA positive) are among HBV-infected patients in the United States. Prior studies in China and France have noted between 3.4 (26), 4.9 (27), and 8.9% (14) of HBV-infected patients to have coexistent HBsAg and anti-HBs. Anti-HBs escape mutations have been described in the USA in infants born to HBV-infected mothers following postnatal HBV vaccine and hepatitis B immune globulin (HBIG) prophylaxis and in many liver-transplant recipients who develop HBV re-infection despite HBIG prophylaxis (28–30) as well as one HBV vaccinated patient post lung transplant on hemodialysis (31). To the best of our knowledge, this is the first report of a mutation in the “a”-determinant region (G145A) in a patient in the USA with chronic HBV infection in the absence of receiving HBV vaccine or HBIG (28, 31, 32). G–A substitution leads to a 50% loss of a-determinant and infectivity *in vitro*, while the more common G–R substitution leads to a loss of a-determinant but a gain in infectivity *in vitro*, explaining why this G145R preferentially emerges (20, 33). The positive charge of R enhances attachment to cell surface heparan sulfate and hence infectivity.

This case is significant because it highlights the potential need for a greater sequence surveillance of populations outside those previously known to be high risk for escape mutations (transplant patients and infants born to HBV-infected mothers).

In addition to immune escape mutation, the HBV sequence analysis in this case revealed HBV genotype C1 with a small deletion in preS2. PreS2 deletion mutations have been previously described especially in Asian patients with HBV genotype C (2) and are associated with a higher likelihood of hepatocellular carcinoma (HCC) in both adults and children, as well as the coexistence of HBsAg and anti-HBs (34–36). Mutations in the HBV pre-S2 region have been demonstrated *in vitro* to cause transactivation of reporter genes, which is thought to contribute to the development of HCC (37, 38). Subsequent studies have determined that preS2 deletion mutations are common in patients with HCC, compared to HBV-infected patients without HCC (39) and that preS2 deletions are associated with the histological progression of ground-glass hepatocytes (40). Ground-glass hepatocytes have been associated with HBV-related fibrosis and HCC (41, 42). Proposed models through which preS2 deletion might cause hepatic pathology include (1) the loss of an HBV epitope of cytotoxic T lymphocytes, favoring viral escape from host immune attack; (2) an increased endoplasmic reticulum (ER) stress, due to accumulation of intracellular envelope proteins (43), contributing to carcinogenesis; and/or (3) the upregulation of cyclin A and cyclooxygenase which decreases the distance between S promoter and transcription initiation site of S mRNA, changing the ratios between the 3 HBV envelope proteins (L-/M-/S-HBsAg) and also increasing ER stress (44). It is notable that the previously described pre-S2 deletions are larger than the two amino acid deletions described here. However, the LY dipeptide is well conserved in all HBV genotypes; therefore, the mutation observed here may point to a crucial importance of LY. There are currently no guidelines for enhanced HCC monitoring among patients with preS2 deletions (of any size).

It is important to note that the combination of “protective” anti-HBs and negative HBsAg observed in this case could have provided false reassurance of HBV immune control and had anti-HBc and subsequent HBV DNA not been checked. The presence of positive anti-HBc prompted HBV DNA testing and sequencing, which revealed important mutations and HBV reactivation during treatment. Had this case progressed to fulminant HBV reactivation, the risk of transmission to vaccinated persons, such as health-care workers, was potentially high, given the potential coexistence of vaccine escape mutations and enhanced replication mutations described. This case underscores the importance of checking anti-HBc in all at-risk patients and subsequently monitoring HBV viral load, especially during HCV treatment. The frequency at which HBV viral load should be monitored, especially during and after HCV clearance and other immune-disruptive events, is not clear. Our current practice is to check HBV DNA every 2–4 weeks during HCV treatment of anti-HBc-positive patients. Finally, this case raises the question of when HBV sequence analysis should be performed and how those data could be centrally managed. HBsAg-negative anti-HBs escape mutants, if transmitted, could undermine vaccine efforts and also go unnoticed without appropriate evolution of diagnostic tests (45, 46).

Furthermore, there is evolving evidence that HBsAg variants may influence HBV treatment response (47).

The potential for this problem should be anticipated, a highly curated central repository for HBV-mutant strains be initiated, and strategy for vaccine adaptation be in place. Roque-Afonso et al. point out that “understanding the prevalence of potential antigenic variation of the HBsAg is fundamental for assay design and to future changes in vaccine formulation” (48). Similarly, Kim et al. suggest that the monitoring of mutation types in GTc with nested PCR (especially in occult cases) “should be imperative for appropriate control of HBV…because…certain mutations in HBV pre-S/-S region might alter (1) HBsAg antigenicity or (2) secretion capacity leading to immune escape infections and eventually emergence of HBV variants in the vaccinated population” (44). Overall, this case underscores the message of Roque-Afonso and Kim and highlights the importance of anti-HBc testing in all at-risk patients, the need for an active surveillance program for HBV sequences, and the importance of ongoing interpretation and anticipation of potential HBV mutation effects on vaccine and antiviral efficacy.

The findings and conclusions in this report are those of the authors and do not necessarily represent the official position of the Centers for Disease Control and Prevention.

## Ethics Statement

This study was carried out in accordance with the recommendations of the Partners Institutional Review Board with written informed consent from the subject. The subject gave written informed consent in accordance with the Declaration of Helsinki. The protocol was approved by the Partners Institutional Review Board.

## Author Contributions

DF, GJ, CS, KA, RC, and GL evaluated the clinical case and contributed to the description of the case evolution. DF, YK, LP, LG, AM, SC, PK, GJ, and CS evaluated the HBV sequence results and contributed to HBV sequence literature review. All authors contributed to the manuscript preparation.

## Conflict of Interest Statement

The authors declare that the research was conducted in the absence of any commercial or financial relationships that could be construed as a potential conflict of interest.

## Supplementary Material

Table S1 Literature review of hepatitis B virus reactivation during hepatitis C virus treatment.

Table S2 Literature review of hepatitis B virus vaccine escape mutants.
