# Supplementary material for: Reactivation of a Vaccine Escape Hepatitis B Virus Mutant in a Cambodian Patient During Anti-Hepatitis C Virus Therapy
Source: Front Med (Lausanne). 2018 Apr 30;5:97. doi: 10.3389/fmed.2018.00097 (PMC5936758; doi:10.3389/fmed.2018.00097)
Supplement: Table S1 — Literature review of hepatitis B virus reactivation during hepatitis C virus treatment. [file Table_1.DOCX]

Supplements

Table S1

| **Author** | **Year** | **Title** | **Treatment** |
| --- | --- | --- | --- |
| Wahle | 2015 | Hepatitis B virus reactivation after treatment for hepatitis C in hemodialysis patients with HBV/HCV coinfection | interferon-alpha 3 MU 3×/week for 12 months |
| Fabbri | 2017 | Reactivation of occult HBV infection in an HIV/HCV Co-infected patient successfully treated with sofosbuvir/ledipasvir: a case report and review of the literature. | sofosbuvir/ledipasvir |
| Balagopal | 2015 | Editorial Commentary: Another Call to Cure Hepatitis B. | Collins Study- sofosbuvir and simeprevir |
| Liu | 2014 | Treatment of patients with dual hepatitis C virus and hepatitis B virus infection: resolved and unresolved issues. |  |
| Uyanikoglu | 2013 | Co-infection with hepatitis B does not alter treatment response in chronic hepatitis C. | Patients who were diagnosed before 2001 received standard interferon/ribavirin, and those after 2001 received pegylated  interferon/ribavirin combination therapy. |
| Raheem | 2013 | Treatment of hepatitis B virus reactivation in a cadaveric renal transplant recipient with entecavir. | Entecavir |
| Kim | 2011 | Clinical features and treatment efficacy of peginterferon alfa plus ribavirin in chronic hepatitis C patients coinfected with hepatitis B virus. | pegylated interferon alfa-2a plus ribavirin or pegylated interferon alfa-2b plus ribavirin. |
| Hung | 2012 | Precore/core promoter mutations and hepatitis B virus genotype in hepatitis B and C dually infected patients treated with interferon-based therapy. | IFN or pegylated-IFN plus ribavirin |
| Yu | 2010 | HBsAg profiles in patients receiving peginterferon alfa-2a plus ribavirin for the treatment of dual chronic infection with hepatitis B and C viruses. | Peginterferon Alfa-2a plus Ribavirin |
| Polilli | 2010 | Fatal reactivation of HBV and HDV during a long-lasting interruption of HAART in a patient co-infected with HIV, HCV, HBV and HDV. | pegylated interferon |
| Viganò | 2009 | The course of inactive hepatitis B in hepatitis-C-coinfected patients treated with interferon and ribavirin. | RBV with either standard IFN (n=14) or pegylated (PEG)-IFN-alpha2b |
| Saitta | 2006 | Virological profiles in hepatitis B virus/hepatitis C virus coinfected patients under interferon plus ribavirin therapy. | interferon plus ribavirin |
| Yalcin | 2003 | A severe hepatitis flare in an HBV-HCV coinfected patient during combination therapy with alpha-interferon and ribavirin. | α-IFN plus ribavirin therapy, |
| Holmes | 2017 | Hepatitis B reactivation during or after direct acting antiviral therapy - implication for susceptible individuals. |  |
| Bersoff-Matcha | 2017 | Hepatitis B Virus Reactivation Associated With Direct-Acting Antiviral Therapy for Chronic Hepatitis C Virus: A Review of Cases Reported to the U.S. Food and Drug Administration Adverse Event Reporting System. | second-generation DAA |
| Hayashi | 2016 | A case of acute hepatitis B in a chronic hepatitis C patient after daclatasvir and asunaprevir combination therapy: hepatitis B virus reactivation or acute self-limited hepatitis? | daclatasvir and asunaprevir therapy |
| Ende AR | 2015 | Fulminant hepatitis B reactivation leading to liver transplantation in a patient with chronic hepatitis C treated with simeprevir and sofosbuvir: a case report | Simeprevir and sofosbuvir |
| Wang | 2017 | Hepatitis due to Reactivation of Hepatitis B Virus in EndemicAreas Among Patients With Hepatitis C Treated With Direct-acting Antiviral Agents. |  |
| Collins | 2015 | Hepatitis B Virus Reactivation During Successful Treatment of Hepatitis C Virus With Sofosbuvir and Simeprevir. | sofosbuvir and simeprevir |
| De Monte | 2016 | Direct-acting antiviral treatment in adults infected with hepatitis C virus: Reactivation of hepatitis B virus coinfection as a further challenge. | ledipasvir/sofosbuvir for 12 weeks. |
| Takayama | 2016 | Reactivation of hepatitis B virus during interferon-free therapy with daclatasvir and asunaprevir in patient with hepatitis B virus/hepatitis C virus co-infection | Daclatasvir and asunaprevir therapy |
| Kimura | 2015 | Sustained hepatitis C virus RNA clearance accompanied by elevation of hepatitis B virus DNA after short-term peginterferon-α, ribavirin and simeprevir therapy in a chronic hepatitis patient having dual infection with hepatitis B and C viruses |  |
| Ou | 2017 | Hepatitis B reactivation in a chronic hepatitis C patient treated with ledipasvir and sofosbuvir: A case report | ledipasvir/sofosbuvir |
