# Supplementary material for: Reactivation of a Vaccine Escape Hepatitis B Virus Mutant in a Cambodian Patient During Anti-Hepatitis C Virus Therapy
Source: Front Med (Lausanne). 2018 Apr 30;5:97. doi: 10.3389/fmed.2018.00097 (PMC5936758; doi:10.3389/fmed.2018.00097)
Supplement: Table S2 — Literature review of hepatitis B virus vaccine escape mutants. [file Table_2.DOCX]

Table S2

| **Author** | **Year** | **Title** | **Mutation** |
| --- | --- | --- | --- |
| Zanetti | 1988 | [Hepatitis B variant in Europe.](https://www.ncbi.nlm.nih.gov/pubmed/2460710) | G145R (guanosine to adenosine at nucleotide position 587) |
| Carman | 1990 | [Vaccine-induced escape mutant of hepatitis B virus.](https://www.ncbi.nlm.nih.gov/pubmed/1697396) | HBsAg |
| Zhongua | 1994 | [Sequencing of hepatitis B virus DNA fragment coding major HBsAg of escape mutant] | threonine to alanime at the amino acid position 126 of HBsAg. |
| Koff RS | 1994 | Problem hepatitis viruses: the mutants. | No specific mutation mentioned |
| Ni F | 1995 | A new immune escape mutant of hepatitis B virus with an Asp to Ala substitution in aa144 of the envelope major protein. | HBV mutant with envelope major protein144Asp-->Ala |
| Hino K | 1995 | Glycine-to-arginine substitution at codon 145 of HBsAg in two infants born to hepatitis B e antigen-positive carrier | G145R |
| Magnius LO | 1995 | Subtypes, genotypes and molecular epidemiology of the hepatitis B virus as reflected by sequence variability of the S-gene. | review |
| Carman WF | 1996 | Hepatitis B Virus Envelope Variation After Transplantation With and Without Hepatitis B Immune Globulin Prophylaxis | G145R and others |
| Zhong | 1996 | [Identification of vertical transmission of hepatitis B virus from mother to children by direct sequencing a segment of surface gene of hepatitis B virus]. | G145R |
| Ngui | 1998 | [Failed postnatal immunoprophylaxis for hepatitis B: characteristics of maternal hepatitis B virus as risk factors.](https://www.ncbi.nlm.nih.gov/pubmed/9675462) | C158, A328, G365, and A479 |
| Ghany MG | 1998 | Hepatitis B virus S mutants in liver transplant recipients who were reinfected despite hepatitis B immune globulin prophylaxis. | G145R,Y134F,P142S,D144A/E,S143T,T126A,P127S/T,125M,T131N |
| Protzer-Knolle U | 1998 | Interleukin-10 expression is autoregulated at the transcriptional level in human and murine Kupffer cells. | sX144G and sG145A, "escape" variants s144 or s145 |
| Yeh CT | 2000 | dentification and characterization of a prevalent hepatitis B virus X protein mutant in Taiwanese patients with hepatocellular carcinoma. | HBx-A31 mutant |
| Bock CT | 2000 | The enhancer I core region contributes to the replication level of hepatitis B virus in vivo and in vitro. | mutations in the enhancer I region |
| Cooreman | 2001 | [Vaccine- and hepatitis B immune globulin-induced escape mutations of hepatitis B virus surface antigen.](https://www.ncbi.nlm.nih.gov/pubmed/11385295) | review, G145R |
| He | 2001 | Prevalence of vaccine-induced escape mutants of hepatitis B virus in the adult population in China: a prospective study in 176 restaurant employees. | HBSAg +, Gly-145-Ala |
| Webber | 2001 | Hepatitis B virus markers in anti-HBc only positive individuals | escape mutant: anti HBc, HBsAg |
| Cooreman | 2001 | Vaccine- and hepatitis B immune globulin-induced escape mutations of hepatitis B virus surface antigen. | HBsAg, G145R |
| Nainan | 2002 | Genetic variation of hepatitis B surface antigen coding region among infants with chronic hepatitis B virus infection. | G145R |
| Oon | 2002 | Molecular characterization of hepatitis B virus surface antigen mutants in Singapore patients with hepatocellular carcinoma and hepatitis B virus carriers negative for HBsAg but positive for anti-HBs and anti-HBc. | Mutations on the a-determinant of hepatitis B virus surface antigen (HBsAg) |
| [Chakravarty](https://www-ncbi-nlm-nih-gov.ezp-prod1.hul.harvard.edu/pubmed/?term=Chakravarty%20R%5BAuthor%5D&cauthor=true&cauthor_uid=12457969) | 2002 | Presence of hepatitis B surface antigen mutant G145R DNA in the peripheral blood leukocytes of the family members of an asymptomatic carrier and evidence of its horizontal transmission. | G145R |
| Lu | 2003 | De novo infection in a renal transplant recipient caused by novel mutants of hepatitis B virus despite the presence of protective anti-hepatitis B surface antibody. | HBsAg a-determinant |
| [Locarnini](https://www-ncbi-nlm-nih-gov.ezp-prod1.hul.harvard.edu/pubmed/?term=Locarnini%20S%5BAuthor%5D&cauthor=true&cauthor_uid=12616447) | 2003 | The hepatitis B virus and common mutants. | BCP mutation: A1762T plus G1764A;double mutation of A1762T plus G1764A results in a decrease in HBeAg production of up to 70% |
| Hsu | 2004 | [Interferon-Based Treatment of Hepatitis C Virus Infection Reduces All-Cause Mortality in Patients With End-Stage Renal Disease: An 8-Year Nationwide Cohort Study in Taiwan.](https://www.ncbi.nlm.nih.gov/pubmed/26632730) | G145R. HBS Ag |
| [Louisirirotchanaku](https://www-ncbi-nlm-nih-gov.ezp-prod1.hul.harvard.edu/pubmed/?term=Louisirirotchanakul%20S%5BAuthor%5D&cauthor=true&cauthor_uid=15357910) | 2004 | Mutation of the "a" determinant of HBsAg with discordant HBsAg diagnostic kits. | G145R |
| Huy TT | 2004 | Characteristics of core promoter and precore stop codon mutants of hepatitis B virus in Vietnam | CP/PC mutants : PC mutant (A1896) and CP mutants, 1762/A1764 double mutant |
| [Levicnik-Stezinar](https://www-ncbi-nlm-nih-gov.ezp-prod1.hul.harvard.edu/pubmed/?term=Levicnik-Stezinar%20S%5BAuthor%5D&cauthor=true&cauthor_uid=15000220) | 2004 | Hepatitis B surface antigen escape mutant in a first time blood donor potentially missed by a routine screening assay. | blood donor with a mutation so far undescribed that alters the common 'a' determinant of HBsAg in such a way that several immunoassays fail to detect this hepatitis B virus infected patien |
| Santos | 2004 | Hepatitis B virus variants in an HIV-HBV co-infected patient at different periods of antiretroviral treatment with and without lamivudine. | V519L, L526M, M550V |
| Pawlotsky | 2005 | The concept of hepatitis B virus mutant escape | HBV exists as quasispecies |
| Mascagni | 2005 | [Characterisation of an HBsAg mutant of hepatitis B virus (HBV) isolated from a dialysed patient involved in an occupational accident]. | Article in italian: threonine to lysine substitution at position 118 of HBsAg (Thrll8Lys) |
| [Roque-Afonso](https://www.ncbi.nlm.nih.gov/pubmed/?term=Roque-Afonso%20AM%5BAuthor%5D&cauthor=true&cauthor_uid=16099111) | 2005 | [HBs antigen mutants: prevalence, clinical and diagnostic implications]. | Changes I195 M and W196L, reflecting lamivudine resistance are present among 18 patients (33%). The most frequent substitutions involved the following amino-acids: F/Y134 (7/31), M133 (6/31), D144 (6/31) and G145 (6/31). |
| Hu | 2005 | [Detection of mutants of the "a" determinant region of hepatitis B surface antigen S gene among Wuhan childhood patients | Mutations of the "a" determinant |
| Lee | 2005 | Overlapping gene mutations of hepatitis B virus in a chronic hepatitis B patient with hepatitis B surface antigen loss during lamivudine therapy. | pre-S2 deletions and 'a' determinant substitutions with YMDD mutation |
| [Margeridon](https://www-ncbi-nlm-nih-gov.ezp-prod1.hul.harvard.edu/pubmed/?term=Margeridon%20S%5BAuthor%5D&cauthor=true&cauthor_uid=15914846) | 2005 | A quasi-monoclonal anti-HBs response can lead to immune escape of 'wild-type' hepatitis B virus. | Pt. with HBV of genotype C and serotype adr modified by a point mutation to express HBsAg of serotype ayr |
| Liu | 2005 | Hot-spot mutations in hepatitis B virus core gene: eliciting or evading immune clearance? | substitutions L60V and I97L |
| Weber | 2005 | Detection of an acute asymptomatic HBsAg negative hepatitis B virus infection in a blood donor by HBV DNA testing. | G145R |
| Ijaz | 2006 | A low rate of hepatitis B virus vaccine breakthrough infections in Mongolia. | G145A |
| Chang | 2006 | Hepatitis B virus mutation in children. | YMDD mutation of the HBV polymerase gene |
| Mathet | 2006 | Detection of hepatitis B virus (HBV) genotype E carried--even in the presence of high titers of anti-HBs antibodies--by an Argentinean patient of African descent who had received vaccination against HBV. | D144A HBsAg escape mutant and L209V |
| Tabor | 2006 | Infections by hepatitis B surface antigen gene mutants in Europe and North America. | G145R |
| Lada | 2006 | [Coexistence of hepatitis B surface antigen (HBs Ag) and anti-HBs antibodies in chronic hepatitis B virus carriers: influence of "a" determinant variants.](https://secure.med.harvard.edu/pubmed/,DanaInfo=www-ncbi-nlm-nih-gov.ezp-prod1.hul.harvard.edu,SSL+16501106) | changes in group I patients were located at positions s145, s129, s126, s144, and s123, as described for immune escape variants |
| [Avellón](https://www.ncbi.nlm.nih.gov/pubmed/?term=Avell%C3%B3n%20A%5BAuthor%5D&cauthor=true&cauthor_uid=16299725) | 2006 | Frequency of hepatitis B virus 'a' determinant variants in unselected Spanish chronic carriers. | 1.5% G145A ; Gly145Arg (0.4%) ; 6.6% overall prevalence of vaccine escape mutations in 272 chronic hep b pts |
| [Huy](https://www.ncbi.nlm.nih.gov/pubmed/?term=Huy%20TT%5BAuthor%5D&cauthor=true&cauthor_uid=16372296) | 2006 | Characteristics of hepatitis B virus in Ghana: full length genome sequences indicate the endemicity of genotype E in West Africa. | G145R escape mutant |
| Amini-Bavil-Olyaee S | 2010 | [Differential impact of immune escape mutations G145R and P120T on the replication of lamivudine-resistant hepatitis B virus e antigen-positive and -negative strains.](https://www.ncbi.nlm.nih.gov/pubmed/19889778) | sG145R mutation, reduced HBsAg, PC or BCP mutation |
| Sheldon | 2010 | Mutations affecting the replication capacity of the hepatitis B virus. | sG145R, sP120T --> rtL180M + rtM204I (see Table 1) |
| Ni | 2011 | Natural history of hepatitis B virus infection: pediatric perspective. | review |
| Lai | 2012 | Increased seroprevalence of HBV DNA with mutations in s gene among individuals> 18 years old after complete vaccination. | inside the major hydrophilic region (MHR) : sS114T, sT131N, sG145A, sE164V and outside the MRH: sR78Q, sL173F,sL186P, sI195M, sY200H, sL216*, sW223* |
| Foy | 2012 | [False-negative hepatitis B virus (HBV) surface antigen in a vaccinated dialysis patient with a high level of HBV DNA in the United States.](https://www.ncbi.nlm.nih.gov/pubmed/22441395) | HBSAg |
| Sa-Nguanmoo | 2012 | [Molecular analysis of hepatitis B virus associated with vaccine failure in infants and mothers: a case-control study in Thailand.](https://www.ncbi.nlm.nih.gov/pubmed/22711345) | a determinant region (residues 144 and 145) |
| Sticchi | 2013 | [Epidemiology of HBV S-gene mutants in the Liguria Region, Italy: Implications for surveillance and detection of new escape variants.](https://www.ncbi.nlm.nih.gov/pubmed/23296324) | Italy: G145R mutation in 8/256 (3.1%) examined sequences; 3 samples with HB Sag undetectable (T126I-T131A-C139Y-E/D144G, T126I-M133L, and P120Q-T126I) |
| Lin | 2013 | Naturally occurring hepatitis B virus B-cell and T-cell epitope mutants in hepatitis B vaccinated children. | Taiwan: G145A |
| Lacombe | 2013 | High incidence of treatment-induced and vaccine-escape hepatitis B virus mutants among human immunodeficiency virus/hepatitis B-infected patients. | s120/s145, 6.4% |
| Larralde | 2013 | [Hepatitis B escape mutants in Scottish blood donors.](https://www.ncbi.nlm.nih.gov/pubmed/23274404) | P127A/T |
| Ishigami | 2014 | Frequent incidence of escape mutants after successful hepatitis B vaccine response and stopping of nucleos(t)ide analogues in liver transplant recipients. | G145R, T131P, S143T, D144A, S114T, T113S, I126T, others |
| Rodríguez Lay | 2015 | Genetic Diversity of the Hepatitis B Virus Strains in Cuba: Absence of West-African Genotypes despite the Transatlantic Slave Trade. | G145R |
| Coppola | 2015 | [Clinical significance of hepatitis B surface antigen mutants.](https://www.ncbi.nlm.nih.gov/pubmed/26644816) | SEE TABLE 2 for summary of vaccine escape mutants through 2015 |
| Luongo | 2015 | Acute hepatitis B caused by a vaccine-escape HBV strain in vaccinated subject: sequence analysis and therapeutic strategy. | Italy : Q129H |
| Anderson | 2015 | [Molecular characterisation of hepatitis B virus in HIV-1 subtype C infected patients in Botswana.](https://www.ncbi.nlm.nih.gov/pubmed/26268355) | escape mutants at positions 100, 119, 122, 123, 124, 126, 129, 130, 133, 134 and 140 of the S ORF |
| Wang | 2015 | Serologic and molecular characteristics of hepatitis B virus infection in vaccinated schizophrenia patients in China. | I126S, T118K, P120S, S143L, Q129H, G145A, P120T, T126N, G145R |
| Ye | 2015 | A new vaccine escape mutant of hepatitis B virus causes occult infection. | Pro 120 Gln, Glu 164 Gly, S171F, S174N, Q181R |
| [Amponsah-Dacosta](https://secure.med.harvard.edu/pubmed/,DanaInfo=www-ncbi-nlm-nih-gov.ezp-prod1.hul.harvard.edu,SSL+?term=Amponsah-Dacosta%20E%5BAuthor%5D&cauthor=true&cauthor_uid=25600597) | 2015 | Hepatitis B virus infection in post-vaccination South Africa: occult HBV infection and circulating surface gene variants. | no mutations |
| Lusida | 2016 | Current hepatitis B virus infection situation in Indonesia and its genetic diversity | T140I,P120S, T126I, M133T/L, T140I, C147S, and S155F |
| Aghasadeghi | 2016 | [Low prevalence of hepatitis B vaccine escape mutants among individuals born after the initiation of a nationwide vaccination program in Iran.](https://www.ncbi.nlm.nih.gov/pubmed/27613286) | I195M; G145R |
| Tong | 2016 | Overview of hepatitis B viral replication and genetic variability. | G145R, T118K, K141E, D144G, C147R, and C149R |
| Salpini | 2016 | [High Burden of HBV-Infection and Atypical HBV Strains among HIV-infected Cameroonians.](https://www.ncbi.nlm.nih.gov/pubmed/26419862) | G145R Cameroon |
| Aghakhani | 2016 | [Occult hepatitis B virus infection and S gene escape mutants in HIV-infected patients after hepatitis B virus vaccination.](https://www.ncbi.nlm.nih.gov/pubmed/26384943) | G145R Iran |
| Al-Qudari | 2016 | Surface gene variants of hepatitis B Virus in Saudi Patients. | amino acids: F130L and S135F,no evidence G145R |
| Sayan | 2016 | Atypical serological profiles in hepatitis B infections: investigation of S gene mutations in cases with concurrently positive for HBsAg and anti-HBs | S143L with sS193L, a HBV vaccine escape mutation, and the other was sP120R, a HBV immune escape mutation |
