# Supplementary material for: Reactivation of a Vaccine Escape Hepatitis B Virus Mutant in a Cambodian Patient During Anti-Hepatitis C Virus Therapy
Source: Front Med (Lausanne). 2018 Apr 30;5:97. doi: 10.3389/fmed.2018.00097 (PMC5936758; doi:10.3389/fmed.2018.00097)
Supplement: Supplementary file 3 [file Data_Sheet_1.DOCX]

Figure S1 Sequence

>MGHHBV

CTCCAGCACATTCCACCGAGCTCAGCTAGATCCCAGAGTGAGGGGCTTTCCTGCTGGTGGCTCAAGTTCCGGGACAGTAAACCCTGTTCCGACTACTGCCTCTCCCATATCGTCAATCTTCTCGAGGACTGGGGACCCTGCACCAAATATGGAGAGCATCACATCAGGATCCCTAGGACCCCTGCTCGTGTTACAGGCGGGGTTTTTCTTGTTGACAAGAATCCTCACAATACCACAGAGTCTAGACTCGTGGTGGACTTCTCTCAATTTTCTAGGGGGAGCACCCACGTGTCCTGGCCAAAATTTGCAGTCCCCAACCTCCAATCACTCACCAACCTCTTGTCCTCCAATTTGTCCTGGTTATCGCTGGATGTGTCTGCGGCGTTTTATCATATTCCTCTTCATCCTGCTGCTATGCCTCATTTTCTTGTTGGTTCTTCTGGACTACCAAGGTATGTTGCCCGTTTGTCCTCTAATTCCAGGAACAACAACTACCAGCATGGGAACATGCAAGGCCTGCACGACTCCTGCTCAAGGAACCTCTATGTTTCCCTCTTGTTGCTGTACAAAAACTTCGGACGCAAATTGCACCTGTATTCCCATCCCATCATCTTGGGCTTTCGCAAGATTCCTATGGGAGCGGGCCTCAGTCCGTTTCTCCTGGCTCAATTTACTAGTGCCATTTGTTCAGTGGTTCGTAGGGCTTTCCCCCATTGTTTGGCTTTCAGTTATATGGATGATGTGGTATTGGGGGCCAAGTCTGTACAACATCTTGAATCCCTTTTTACCTCTATTACCAATTTTCTTTTATCTTTGGGTATACATTTAAACCCTAATAAAACCAAACGTTGGGGGTACTCCCTTAACTTCATGGGATATGTAATTGGTAGTTGGGGTACCTTGCCACAGGAACATATTGTACTAAAATTGAAACAATGTTTTCGGAAACTTCCTACAAATAGGCCTATTGATTGGAAAGTATGTCAACGAATTGTGGGTCTTCTGGGCTTTGCCGCTCCTTTTACACAATGTGGCTACCCAGCATTAATGCCTTTATATGCATGTATACAAGCTAAGCAGGCTTTCACTTTCTCGCCAACTTATAAGGCCTTTCTGTGTAAACAATATCTGAACCTTTACCCCGTTGCTCGGCAACGGTCGGGTCTTTGCCAAGTGTTTGCTGACGCAACCCCCACTGGTTGGGGCTTGGCCATGGGCCATCAGCGCATGCGTGGAACCTTTGTGGCTCCTCTGCCGATCCATACTGCGGAACTCCTAGCAGCTTGTTTTGCTCGCAGCCGGTCTGGAGCAAAACTTATCGGCACCGACAACTCTGTTGTCCTCTCTCGGAAATACACCTCCTTTCCAAGCCTCCAAGCTGTGCTGCCAACTGGATCCTGCGCGGGACGTCCTTTGTCTACGTCCCGTCGGCGCTGAATCCCGCGGACGACCCCTCTCGGGGCCGGTTGGGGCTCTACCGTCCCCTTCTTCGTCTGCCGTTCCGACCGACCACGGGGCGCACCTCTCTTTACGCGGTCTCCCCGTCTGTGCCTTCTCATCTGCCGGACCGTGTGGATTCGCTTCACCTCTGCACGTCGCATGGAGACCACCGTGAACGCCCGCCAAGTCTTGCCCAAGGTCTTACATAAGAGGACTCTTGGACTCCCAGCAATGTCAACGACCGACCTTGAGGCATACTTCAAAGACTGTGTATTTAAAGACTGGGAGGAGTTGGGGGAGGAGACCAGGTTAATGATCTTTGTACTAGGAGGCTGTAGGCATAAATTGGTCTGCTCACCAGCACCATGCAACTTTTTCACCTCTGCCTAATCATCTCATGTTCATGTCCTACTGTTCAAGCCTCCAAGCTGTGCCTTGGGTGGCTTTGGGGCATGGACATTGACCCGTATAAAGAATTTGGAGCTTCTGTGGAGTTACTCTCTTTTTTGCCTTCTGACTTCTTTACGTCTATTCGAGATCTCCTCGACACCGCCTCTGCTCTGTATCGGGAGGCCTTAGAGTCTCCGGAACATTGTTCAGCTCACCATACAGCACTAAGGCAAGCTATTTTGTGTTGGGGTGAGTTGATGAATCTGGCCACCTGGGTGGGAGGTAATTTGGAAGACCCAGCATCCAGGGAATTAGTAGTAAGCTATGTCAATGTTAACATGGGCCTAAAACTCAGACAAATATTGTGGTTTCACATTTCCTGTCTTACTTTTGGAAGAGAAACTGTTCTAGAGTATTTGGTGTCTTTTGGAGTGTGGATTCGCACTCCTACCGCTTACAGACCACCAAATGCCCCTATCTTATCAACACTTCCGGAGATTACTGTTGTTAGACGACGAGGCAGGTCCCCTAGAAGAAGAACTCCCTCGCCTCGCAGACGAAGATCTCAATCGCCGCGTCGCAGAAGATCTCAATCTCGGGAATCTCAATGTTAGTATCCCTTGGACTCATAAGGTGGGAAACTTTACGGGACTTTATTCTTCTACTGTACCTGTCTTTAATCCTGACTGGCAAACTCCCTCTTTTCCTAACATTCATTTGAAAGAGGATATTATCAACAGATGTCAACAATTTGTAGGCCCTCTTACAGTTAATGAAAAAAGGAGATTAAAATTGATTATGCCTGCTAGGTTCTATCCTAACCTTACCAAATATTTGCCCTTAGATAAAGGTATTAAACCTTATTATCCTGAACATTCAGTTAATCATTACTTCCAAACCAGACATTATTTACATACTCTGTGGAAGGCTGGTATTTTATATAAGAGAGAAACTACACGCAGTGCCTCATTTTGTGGGTCACCATATTCTTGGGAACAAGAGCTACAGCATGGGAGGTTGGTCTTCCAAACCTCGACAAGGCATGGGGACGAACCTTTCTGTTCCCAATCCTCTGGGATTCTTTCCCGGTCACCAGTTGGACCCAGCGTTCAGAGCCAATTCAAACAATCCAGATTGGGACTTCAACCCCAACAAGGATCAATGGCCAGCGGCAAACCAGGTAGGAGTGGGGTCATTCGGGCCAGGGTTCACTCCACCACACGGCAGTCTTTTGGGGTGGAGCCCTCAGGCTCAGGGCACATTGACAACAGTGCCAGCAGCGCCTCCTCCTGCCTCCACCAATCGGCAGTCAGGAAGAAAGCCTACTCCCATCTCTCCACCTCTAAGAGACAGTCATCCTCAGGCCATGCAGTGGAA
