# Supplementary material for: Reactivation of a Vaccine Escape Hepatitis B Virus Mutant in a Cambodian Patient During Anti-Hepatitis C Virus Therapy
Source: Front Med (Lausanne). 2018 Apr 30;5:97. doi: 10.3389/fmed.2018.00097 (PMC5936758; doi:10.3389/fmed.2018.00097)
Supplement: Supplementary file 4 [file Data_Sheet_2.DOCX]

Figure S2 Sequence translation

CDS 1806..2444

Core

Translation= MQLFHLCLIISCSCPTVQASKLCLGWLWGMDIDPYKEFGASVELLSFLPSDFFTSIRDLLDTASALYREALESPEHCSAHHTALRQAILCWGELMNLATWVGGNLEDPASRELVVSYVNVNMGLKLRQILWFHISCLTFGRETVLEYLVSFGVWIRTPTAYRPPNAPILSTLPEITVVRRRGRSPRRRTPSPRRRRSQSPRRRRSQSRESQC

CDS 2840..829 (wrapping)

S gene

MGGWSSKPRQGMGTNLSVPNPLGFFPGHQLDPAFRANSNNPDWDFNPNKDQWPAANQVGVGSFGPGFTPPHGSLLGWSPQAQGTLTTVPAAPPPASTNRQSGRKPTPISPPLRDSHPQAMQWNSSTFHRAQLDPRVRGFPAGGSSSGTVNPVPTTASPISSIFSRTGDPAPNMESITSGSLGPLLVLQAGFFLLTRILTIPQSLDSWWTSLNFLGGAPTCPGQNLQSPTSNHSPTSCPPICPGYRWMCLRRFIIFLFILLLCLIFLLVLLDYQGMLPVCPLIPGTTTTSMGTCKACTTPAQGTSMFPSCCCTKTSDANCTCIPIPSSWAFARFLWERASVRFSWLNLLVPFVQWFVGLSPIVWLSVIWMMWYWGPSLYNILNPFLPLLPIFFYLWVYI

CDS 2299..1437 (wrapping)

Polymerase

MPLSYQHFRRLLLLDDEAGPLEEELPRLADEDLNRRVAEDLNLGNLNVSIPWTHKVGNFTGLYSSTVPVFNPDWQTPSFPNIHLKEDIINRCQQFVGPLTVNEKRRLKLIMPARFYPNLTKYLPLDKGIKPYYPEHSVNHYFQTRHYLHTLWKAGILYKRETTRSASFCGSPYSWEQELQHGRLVFQTSTRHGDEPFCSQSSGILSRSPVGPSVQSQFKQSRLGLQPQQGSMASGKPGRSGVIRARVHSTTRQSFGVEPSGSGHIDNSASSASSCLHQSAVRKKAYSHLSTSKRQSSSGHAVELQHIPPSSARSQSEGLSCWWLKFRDSKPCSDYCLSHIVNLLEDWGPCTKYGEHHIRIPRTPARVTGGVFLVDKNPHNTTESRLVVDFSQFSRGSTHVSWPKFAVPNLQSLTNLLSSNLSWLSLDVSAAFYHIPLHPAAMPHFLVGSSGLPRYVARLSSNSRNNNYQHGNMQGLHDSCSRNLYVSLLLLYKNFGRKLHLYSHPIILGFRKIPMGAGLSPFLLAQFTSAICSVVRRAFPHCLAFSYMDDVVLGAKSVQHLESLFTSITNFLLSLGIHLNPNKTKRWGYSLNFMGYVIGSWGTLPQEHIVLKLKQCFRKLPTNRPIDWKVCQRIVGLLGFAAPFTQCGYPALMPLYACIQAKQAFTFSPTYKAFLCKQYLNLYPVARQRSGLCQVFADATPTGWGLAMGHQRMRGTFVAPLPIHTAELLAACFARSRSGAKLIGTDNSVVLSRKYTSFPSLQAVLPTGSCAGRPLSTSRRR

CDS 1438..1830

X gene

IPRTTPLGAGWGSTVPFFVCRSDRPRGAPLFTRSPRLCLLICRTVWIRFTSARRMETTVNARQVLPKVLHKRTLGLPAMSTTDLEAYFKDCVFKDWEELGEETRLMIFVLGGCRHKLVCSPAPCNFFTSA
